# Anode-Layer Ion Source-Assisted Deposition and Bias Voltage Tuning of CrWNbTaMoVSiN High-Entropy Nanostructured Coatings

**DOI:** 10.3390/nano16181145

**Published:** 2026-09-12

**Authors:** Yan Ling, Shikun Liu, Canxin Tian, Changwei Zou

**Affiliations:** School of Physics Science and Technology, Lingnan Normal University, Zhanjiang 524048, China

**Keywords:** high-entropy nanostructured coatings, magnetron sputtering, friction and wear, corrosion resistance performance

## Abstract

In extreme working conditions such as deep-sea and aerospace, failure due to friction and wear of key metal components is very frequent. Traditional binary or ternary nitride protective coatings are unable to achieve a coordinated improvement in terms of hardness, wear resistance, and structural stability. In this manuscript, a high-entropy composite nano-coating of CrWNbTaMoVSiN with an alternating structure of metal and nitride layers was prepared by using anode layer ion source-assisted magnetron sputtering technology. The influence of substrate bias voltage on the microstructure, phase structure, surface chemical state, friction and wear properties of coatings was systematically studied. The findings demonstrate that the substrate bias voltage has a substantial impact on the structure and performance of the CrWNbTaMoVSiN coatings. As the bias voltage increases, the coating deposition rate and thickness decrease, while the grain growth morphology gradually transitions from directionally aligned columnar crystals to equiaxed crystals. The primary phase of the coating is a solid solution with a face-centered cubic (FCC) crystal structure. At a bias voltage of 60 V, the coating exhibits a denser surface and cross-sectional microstructure, fewer structural defects, higher hardness, superior wear resistance, and enhanced electrochemical corrosion protection performance. This study provides critical experimental evidence and theoretical insights into the bias-voltage-dependent regulation mechanism of high-entropy nano-multilayer coatings, thereby supporting the process optimization of high-performance protective coatings and their engineering deployment under extreme service conditions.

## 1. Introduction

Key moving parts and load-bearing structural components in deep-sea environments, major engineering equipment, aerospace, and other fields have been in long-term service under extreme working conditions where friction and wear are coupled with electrochemical corrosion [1,2]. Premature failure of component surfaces not only significantly shortens equipment service life and reduces operational reliability but also poses serious safety hazards and incurs huge economic losses. Surface protective coating technology serves as the most cost-effective core technical measure to mitigate the failure of metal components and enhance their service performance [3]. Although traditional binary and ternary nitride coatings such as TiN, CrN, and TiAlN have been widely applied in industry [4,5,6,7,8,9], they are inherently limited by insufficient hardness, poor high-temperature thermal stability, and an inability to simultaneously enhance wear and corrosion resistance [10,11]. High-entropy alloys have broken through the design framework of traditional alloys by relying on four core characteristics: the high-entropy effect [12,13], the hysteresis diffusion effect [14], the lattice distortion effect, and the cocktail effect [15].

Recently, High-entropy nitride composite coatings have received extensive attention. This type of coating combines the structural advantages of high-entropy alloys with the high strength, toughness, and high chemical stability of ceramic phases [16,17], achieving synergistic optimization of ultra-high hardness, excellent wear and corrosion resistance, and high-temperature stability. X. Shi’s research indicates that introducing Si elements into a multi-component transition metal high-entropy system and precisely regulating the microstructure of nanocrystalline composites by controlling their content is an effective approach to simultaneously enhancing the comprehensive protective performance of high-entropy nitride coatings [18]. By constructing nanoscale multilayer TiSiN/Ag structures, Lijun Ma [19] can significantly enhance coating strength and toughness, providing new ideas for the development and structural design of protective coatings in extreme service environments. Magnetron sputtering technology is one of the mainstream processes for preparing high-entropy alloy coatings [20,21], featuring low deposition temperature, strong film-substrate adhesion, and good controllability of coating structure and composition [22,23]. Substrate bias voltage is a core process parameter for regulating the microstructure and service performance of coatings [24,25]. Substrate bias directly affects the nucleation and growth mode, grain size, density, internal stress, and interface structure of the coating by altering the bombardment energy of deposited particles, thereby having a decisive impact on the mechanical, tribological, and corrosion resistance properties of the coating [20,26]. Currently, scholars at home and abroad have conducted extensive research on the bias voltage regulation laws of high-entropy coatings in typical systems such as AlCrNbSiTi, FeCoCrNiAlN, and ZrNbHfTa, confirming the crucial role of substrate bias voltage in customizing the structure and performance of high-entropy coatings [27,28,29,30,31,32]. However, systematic research remains lacking on how substrate bias influences the structural evolution and multi-performance synergistic regulation of seven-element CrWNbTaMoVSiN nanomultilayer structure high-entropy nanocomposite coatings containing Si, which seriously restricts the composition design, process optimization, and engineering application of high-performance high-entropy protective coatings in this system.

In this paper, CrWNbTaMoVSiN high-entropy nano-multilayer coatings with alternating metal layer/nitride layer structures were fabricated by anode layer ion source-assisted magnetron sputtering technology. The influence of substrate bias voltage on the mechanical, tribological, and electrochemical corrosion resistance properties of the coatings was systematically investigated, and the relationship between microstructure evolution induced by bias voltage and coating service performance was revealed. This thesis provides a crucial experimental basis and theoretical support for the process optimization and engineering application of high-entropy protective coatings in extremely harsh service environments.

## 2. Experimental Procedure

### 2.1. Coating Deposition

In this experiment, anode layer ion source-assisted magnetron sputtering technology was employed to deposit high-entropy nano-multilayer coatings on the substrate material using the KYVAC-200CK desktop magnetron sputtering coating system. The target material used was a CrWNbTaMoVSi high-entropy alloy target with a purity of 99.5%. The substrate materials were classified into two types: 1 cm × 1 cm × 0.05 cm N-type (111) crystal direction monocrystalline silicon wafers, which were used for coating microstructure characterization; and 8 K mirror-polished 304 stainless steel sheets of 2 cm × 2 cm × 0.2 cm, used for coating mechanical property testing. Ar (purity 99.99%) served as the working gas, and N_2_ (purity 99.99%) was used as the reaction gas.

The coating preparation employs an alternating deposition process. First, a high-entropy metal layer is deposited, followed by a high-entropy nitride layer, forming one modulation cycle. A total of seven cycles are deposited. The specific deposition parameters are as follows: The power of the magnetic control power supply is 100 W, and the power of the ion source is 35 W. For metal layer deposition, the gas ratio is N_2_:Ar = 0:40, with a deposition time of 40 s. For nitride layer deposition, the gas ratio is N_2_:Ar = 10:40, with a deposition time of 40 s. The total deposition time is 560 s. The base bias voltage is taken as the variable, with five sets of parameters set: −40 V, −60 V, −80 V, −100 V, and −120 V.

### 2.2. Characterization and Performance Testing

The surface morphology, cross-sectional morphology, and wear scar morphology after friction and wear of the coating were observed using SEM. Phase analysis of the coating was conducted via XRD with a Cu target Kα radiation source (λ = 0.15406 nm), tube voltage of 40 kV, tube current of 40 mA, a scanning range of 2θ from 10° to 80°, and a scanning rate of 5°/min. The surface chemical state of the coating samples under an 80 V bias was analyzed using XPS. Argon ion etching was not performed prior to the test to preserve the original surface chemical valence state information. Test data were charge-corrected based on the C1s characteristic peak at 284.81 eV. The coating’s hardness was tested using a micro Vickers hardness tester with a test load of 0.01 kg and a holding time of 10 s. For each sample, three random parallel test points were selected, and the average value was taken as the final hardness result to eliminate testing errors. Reciprocating friction and wear tests were conducted using the MPT-4000 multi-functional material surface performance tester under room temperature and atmospheric conditions. The tribopair consisted of a ceramic ball with a diameter of 6 mm. The test parameters included a load of 5 N, a reciprocating stroke of 5 mm, a rotational speed of 200 r/min, and a duration of 60 min. The coefficient of friction curve was smoothed using the Savitzky–Golay method in OriginPro 2025 software with a window size of 10 points. After the tests, the morphology of the wear marks was observed via SEM to analyze the wear mechanism.

The CHI660E electrochemical workstation was used to test the electrochemical corrosion resistance of the coating in artificial seawater at room temperature. A three-electrode system was employed for the test, with a saturated KCl calomel electrode as the reference electrode, a Pt sheet as the auxiliary electrode, and the stainless steel sample coated with the coating as the working electrode, which had an exposure area of 1 cm^2^. First, a 60 min open-circuit potential (OCP) test was conducted. Once the system potential stabilized, the final open-circuit potential value was recorded. Subsequently, using this stable open-circuit potential as the initial potential, an electrochemical impedance spectroscopy (EIS) test was carried out within a frequency range of 0.01 to 10^5^ Hz and with an AC excitation signal amplitude of 5 mV. Potentiodynamic polarization (Tafel) tests were conducted within a polarization voltage range of −1 to +1 V (vs. SCE) at a scanning rate of 1 mV/s. Each set of tests was repeated three times.

## 3. Results and Discussion

### 3.1. The Influence of Substrate Bias Voltage on the Microstructure of the Coatings

#### 3.1.1. Surface Morphology

**Figure 1:**
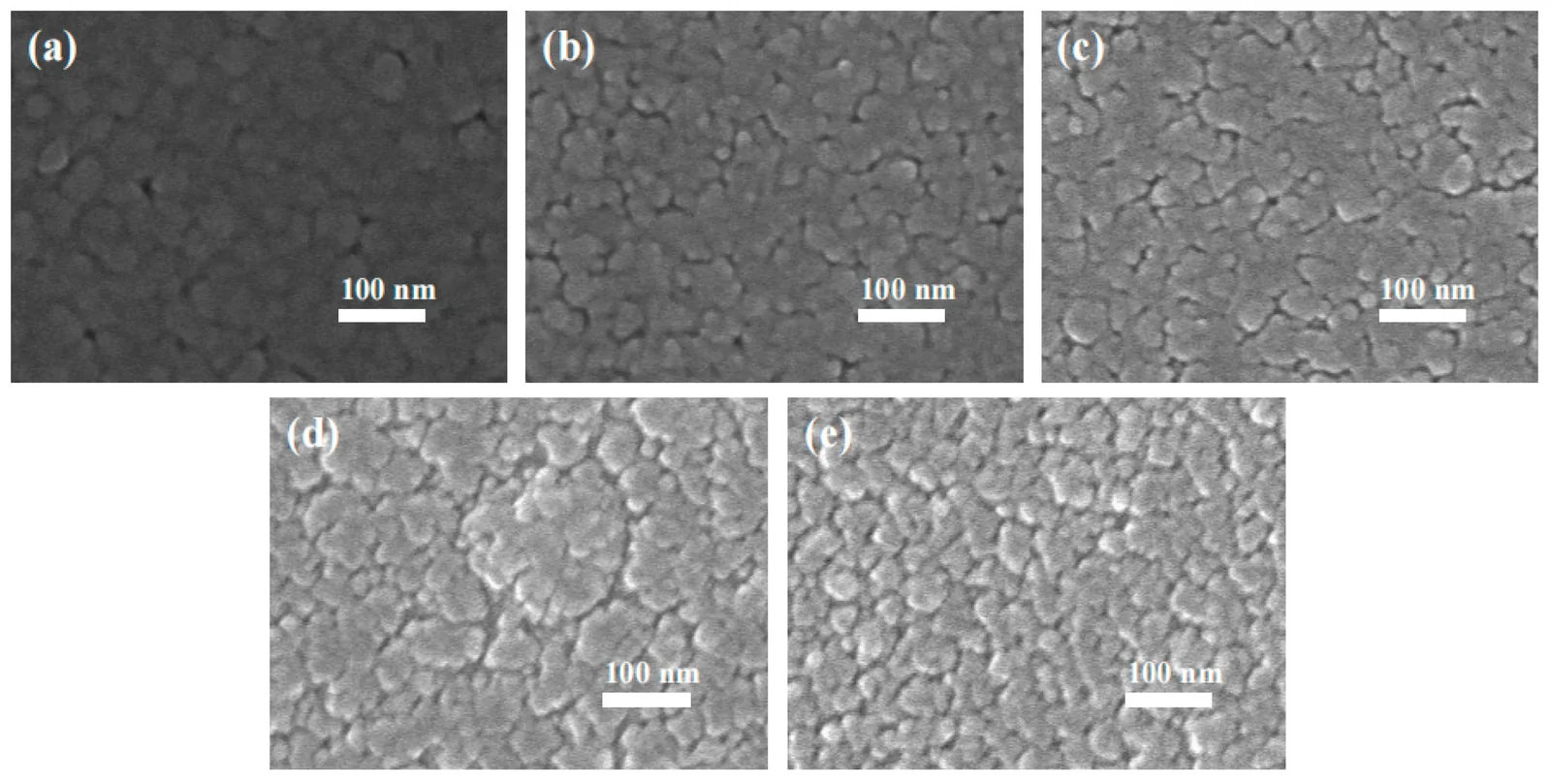
SEM morphology of CrWNbTaMoVSiN coating surface under different substrate bias voltages: (**a**) −40 V. (**b**) −60 V. (**c**) −80 V. (**d**) −100 V. (**e**) −120 V.

The surface SEM morphology of the CrWNbTaMoVSiN high-entropy composite coating under different substrate bias voltages is shown in Figure 1. It can be seen that the substrate bias pressure has a significant regulatory effect on the surface grain size and morphology of the coating. Under a bias voltage of −40 V, the energy of the deposited particles is relatively low, and the atomic surface migration ability is limited. The coating is mainly characterized by uniform nucleation with slow grain growth, resulting in a uniformly dense and refined surface with a grain size of approximately 5–10 nm (Figure 1a). The coating exhibits an extremely low-roughness surface with an overall dense and smooth morphology without obvious holes or protrusions. When the bias voltage rises to −60 to −80 V, the energy of the deposited particles increases, and the nucleation rate and grain growth rate reach a dynamic equilibrium. The grains uniformly grow to 10–15 nm (Figure 1b,c), and the coating surface still maintains good flatness without obvious defects or coarsening. When the bias voltage continues to rise to −100 to −120 V, the particle bombardment energy becomes too high, significantly enhancing the migration ability of atoms on the substrate surface. This leads to grain merging and secondary growth, which significantly coarsens the coating surface grains (size increasing to 20–30 nm, Figure 1d,e). Obvious protrusions and uneven morphology are observed on the coating surface, resulting in a significant increase in roughness and a decrease in the uniformity of coating particles.

#### 3.1.2. Section Morphology and Deposition Rate

**Figure 2:**
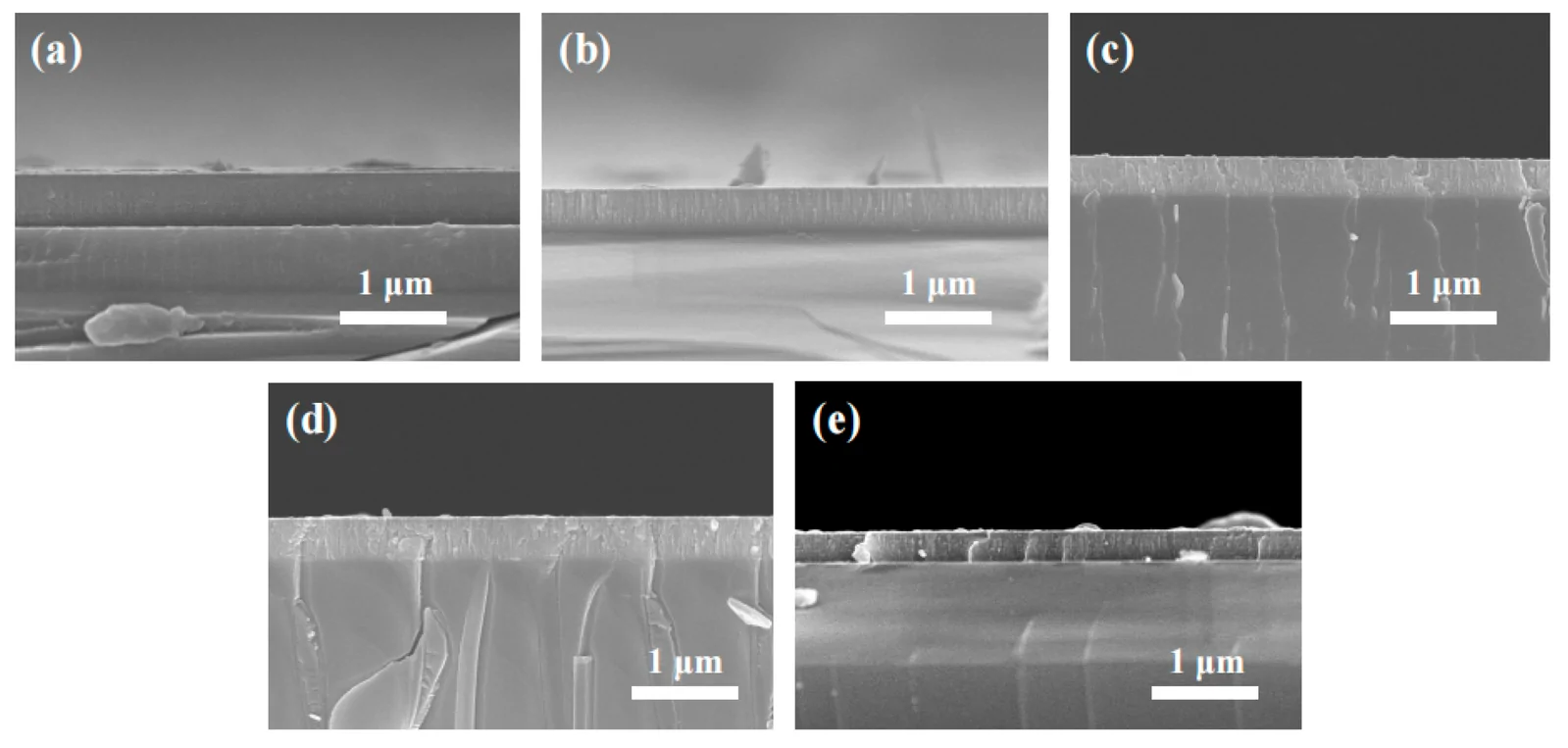
SEM morphology and thickness of CrWNbTaMoVSiN coating cross-section under different substrate bias voltages: (**a**) −40 V; (**b**) −60 V; (**c**) −80 V; (**d**) −100 V; (**e**) −120 V.

The cross-sectional SEM morphologies of the coating under different substrate bias voltages are shown in Figure 2. The results indicate that as the substrate bias voltage increases from −40 V to −120 V, the coating thickness continuously decreases from 538 nm to 353 nm, presenting an approximately linear downward trend. Based on a total deposition time of 560 s, the coating deposition rate decreases from 0.96 nm/s to 0.63 nm/s, indicating that the deposition kinetics process is significantly dependent on the substrate bias pressure.

Under a low bias voltage of −40 V, the coating exhibits a typical dense columnar crystal structure with grains extending directionally perpendicular to the substrate and clear grain boundaries without obvious pores (Figure 2a). This is because the lateral migration ability of deposited particles is limited under low bias voltage, causing atoms to preferentially grow along the normal direction of the substrate and form a typical columnar crystal structure. Additionally, the anti-sputtering effect is weak, and the deposition rate is high under low bias voltage, resulting in the coating having the largest thickness.

When the bias voltage rises to −60 V, the coating thickness drops to 455 nm (Figure 2b), corresponding to a deposition rate of approximately 0.81 nm/s. At this point, the columnar crystal structure still exists, but the grain width has increased. This is because the lateral diffusion ability of particles is enhanced under moderate bias pressure, which has a certain inhibitory effect on the directional growth of columnar crystals. When the bias voltage is increased to −80 V and −100 V, the coating thickness decreases to 412 nm and 388 nm, respectively (Figure 2c,d), with corresponding deposition rates of 0.74 nm/s and 0.69 nm/s. At this stage, the columnar crystal structure gradually disappears, and the coating structure transforms towards densification and refinement, accompanied by the appearance of a small number of micro-pores. This is because under high bias voltage, the strong ion bombardment effect is enhanced, which not only reduces the deposition rate of the coating but also disrupts the directional growth mode of the grains, causing the grain growth direction to shift from vertical to transverse, thus forming a dense and refined structure. When the substrate bias voltage is further increased to −120 V, the coating thickness drops to a minimum of 353 nm (Figure 2e), and the deposition rate decreases to 0.63 nm/s. At this point, the coating shows obvious roughness and microscopic defects. This is due to the excessively high bias voltage, which significantly enhances the sputtering effect. Already deposited surface atoms are sputtered off, greatly reducing the deposition rate. At the same time, the intense ion bombardment introduces a large number of microscopic defects, ultimately forming a thin and rough coating structure. Based on the evolution of surface and cross-sectional morphology, at a bias voltage of 60 to 80V, the coating possesses high density, uniform grain size, and a good interface structure, which is the optimal bias voltage range for preparing the high-entropy composite coating of this system.

**Figure 3:**
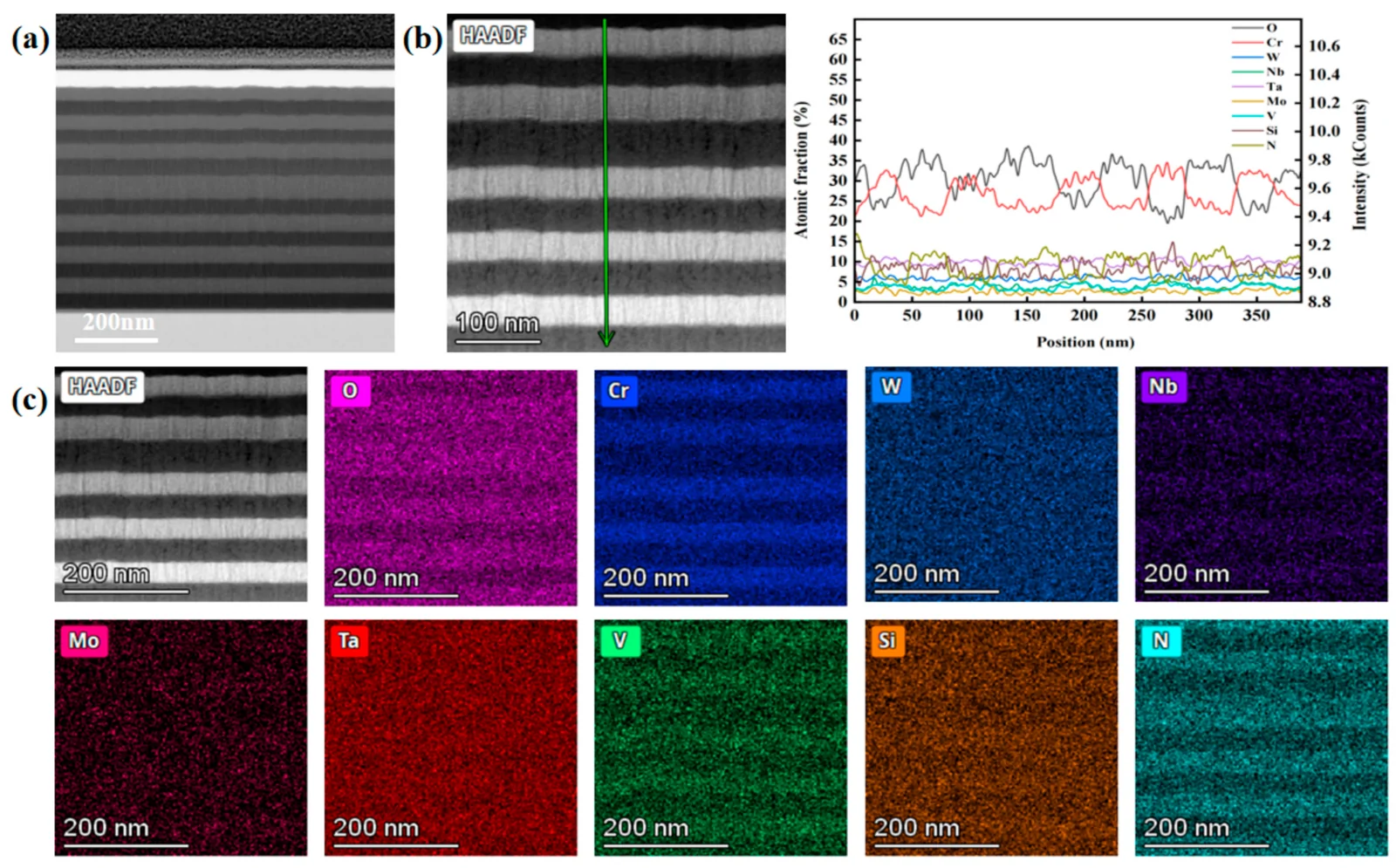
(**a**) The cross-sectional TEM images of films deposited at −60 V. (**b**) EDS line scan spectrum of films deposited at −60 V. (**c**) EDS surface spectrum of films deposited at −60 V.

Figure 3a shows the cross-sectional view of the sample under a transmission electron microscope after FIB treatment at a bias voltage of −60 V. As shown, growth direction along the membrane layer coating in nanometer multilayer structure growth, TEM section morphology to verify the coating for CrWNbTaMoVSi/CrWNbTaMoVSiN periodic structure, modulation periods of about 75 nm, a total of seven periods, coating the total thickness of about 530 nm, the compact structure of coating, and no obvious columnar structure. The interface between the film layer and the hard alloy substrate is clear, without cracks. To further understand the composition of each film in the periodic layer of the nano-multilayer structure, EDS line scanning and surface scanning were carried out on the interface coating, and the concentration of elements was calculated to obtain the depth distribution information of elements in the nano-multilayers. The EDS line scanning is shown in Figure 3b. By carefully distinguishing the depth distribution information of the elements in Figure 3b, the layer with the lighter color is the CrWNbTaMoVSiN layer, and the layer with the darker color is the CrWNbTaMoVSi layer. The two coatings are alternately distributed, with a clear interface, which is consistent with the depth distribution information of the elements shown in the EDS surface scan. Figure 3c. The sample thickness deposited at a −60 V bias measured by SEM is slightly less than the film thickness observed by TEM. The main reason is that the sample is not completely perpendicular to the horizontal plane during SEM cross-sectional sample preparation and observation.

### 3.2. The Influence of Substrate Bias Voltage on the Phase Structure of Coatings

**Figure 4:**
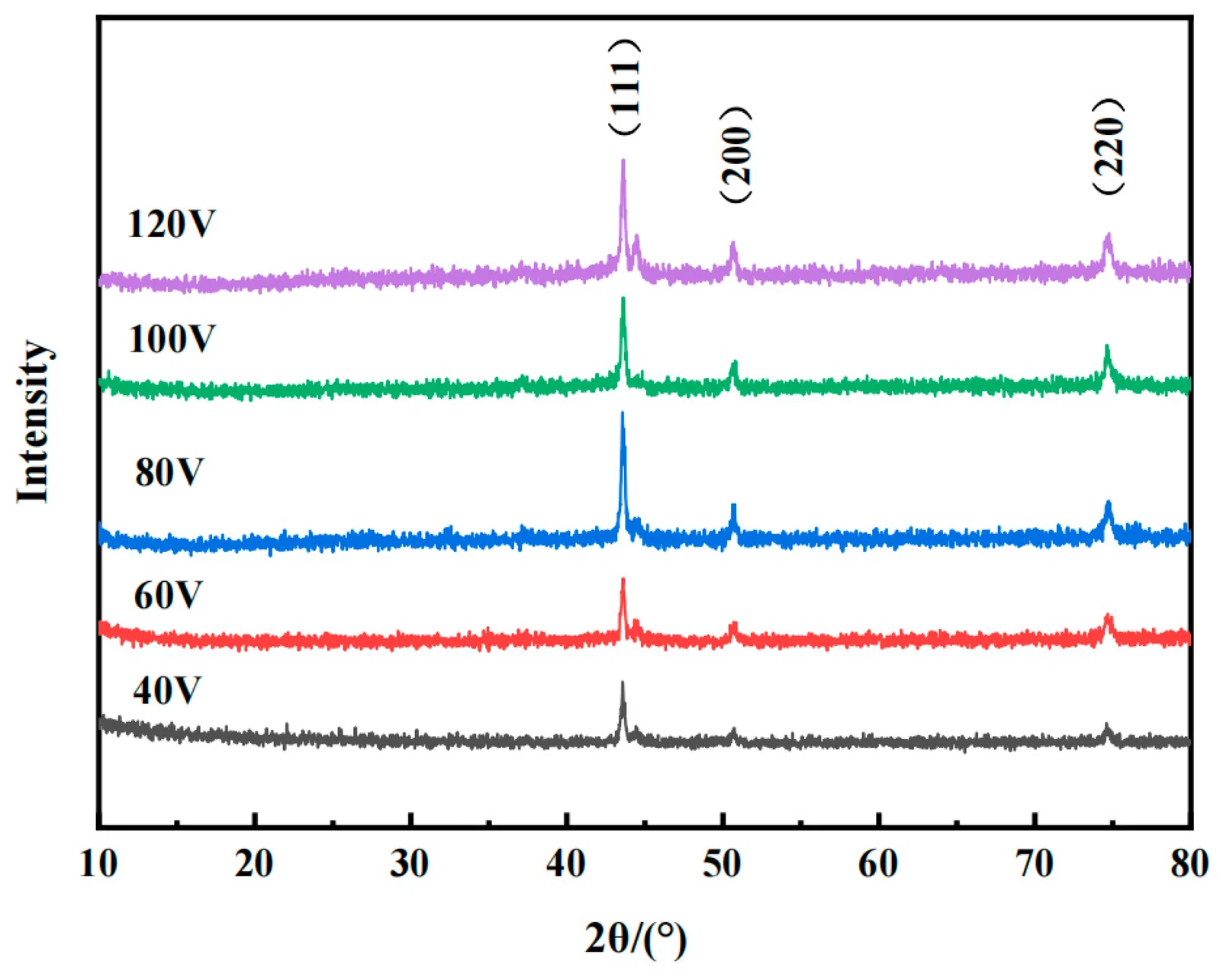
XRD patterns of CrWNbTaMoVSiN high-entropy composite nano-coating under different bias voltages.

The XRD patterns of CrWNbTaMoVSiN high-entropy composite nanocoatings under different bias voltages are shown in Figure 4. A sharp, the high-entropy coatings at different lower levels all have a face-centered cubic structure (FCC). The three diffraction peaks located at 43.5°, 50.7°, and 74.6° in the figure correspond to the (111), (200), and (220) crystal planes in the FCC, respectively. Compared with the diffraction peak of the CrN coating, the diffraction peak of the CrWNbTaMoVSiN high-entropy coating shifts slightly to a smaller angle. This is due to the solid solution of large-radius atoms such as W, Ta, and Mo, forming a displacement solid solution, which leads to lattice distortion and an increase in the lattice constant, causing the diffraction peak to shift to a small Angle [33]. There is also a weak diffraction peak at 44.5° in the figure, which gradually emerges as the bias voltage increases. This might be due to the martensitic transformation peak of the 304 stainless steel substrate. Under high pressure, stronger ion bombardment and a temperature rise are brought about, which will increase the thickness of the phase transformation layer on the substrate surface, leading to the enhancement of this peak. Moreover, from the SEM morphology of the coating cross-section, it can be seen that the higher the bias voltage, the thinner the high-entropy coating. The interference of the substrate on the coating becomes apparent.

### 3.3. Surface Chemical State and Elemental Composition of the Coatings

**Figure 5:**
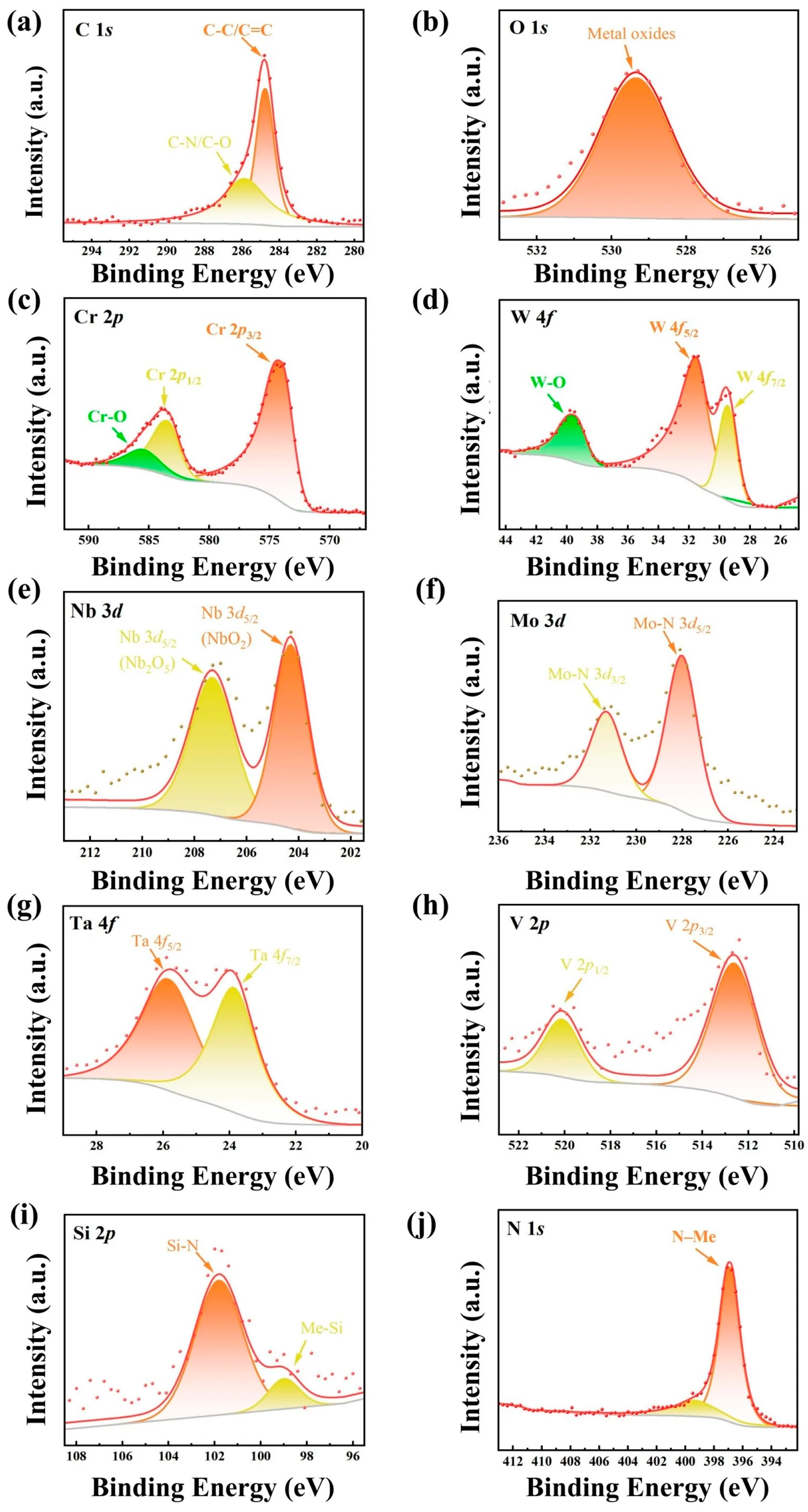
X-ray photoelectron spectroscopy (XPS) of CrWTaMoVSiN high-entropy nano-multilayer coating: (**a**) C1*s*, (**b**) O1*s*, (**c**) Cr2*p*, (**d**) W4*f*, (**e**) Nb3*d*, (**f**) Mo3*d*, (**g**) Ta4*f*, (**h**) V2*p*, (**i**) Si2*p*, (**j**) N1*s*.

To further reveal the chemical state of surface elements of the CrWNbTaMoVSiN high-entropy nanocomposite coatings, high-resolution fine scanning was conducted on the coating prepared under −80V substrate voltage using XPS. No Ar ion etching treatment was performed before the test to retain the true surface chemical information of the coating. Quantitative analysis was conducted based on the peak area and relative sensitivity factor of the narrow-band spectrum [34]. At the same time, it inevitably contains a small amount of surface oxides and adsorbed carbon formed after air exposure, as shown in Figure 5. As shown in the figure, elements such as C, O, Cr, W, Nb, Mo, Ta, V, Si and N can be detected on the surface of the coating, indicating that all the multi-alloy elements are involved in the formation of the chemical structure of the coating surface. After peak fitting of the high-resolution spectra of each element, obvious chemical bonding characteristics were exhibited, indicating that during the reaction magnetron sputtering process, not only multi-metal solid solutions were formed but also nitriding reactions of varying degrees occurred, and a composite chemical structure mainly composed of metal-N bonds was formed.

Figure 5a shows the high-resolution spectrum of C 1*s*, with its main peak located at 284.81 eV. This peak mainly corresponds to the C–C/C=C bond and is also used as a reference peak for binding energy correction. The weak acromion located on the high binding energy side can be attributed to a small amount of C–N or C–O bonds, indicating that there is a certain chemical combination between some non-metallic elements during the deposition process. Furthermore, as the sample has not undergone ion etching treatment, surface adsorption of carbon is inevitable. Therefore, this peak also includes the contribution of polluted carbon formed after air exposure. Overall, the C element does not exhibit obvious characteristics of graphitized carbon but mainly exists in the form of surface-adsorbed carbon and a small amount of chemically bound states.

Figure 5b shows the high-resolution spectrum of O1*s*, with only one relatively wide characteristic peak located near approximately 529.5 eV, which can be attributed to the metal–O bond, indicating that there is a certain degree of oxidation on the coating surface. This result is consistent with the test conditions without ion etching; that is, during contact with air after the coating deposition is completed, elements with high surface activity such as Cr, W, Nb, Mo, Ta and V undergo slight oxidation to form corresponding oxides.

Figure 5c shows the high-resolution spectrum of Cr 2*p*, which presents a typical spin-orbit splitting bipeak structure of transition metal 2*p* orbitals. The main peak of Cr 2*p*_3/2_ is located at 574.60 eV, and Cr 2*p*_1/2_ is approximately 584 eV, with a spin-orbit splitting energy of about 9.6 eV. Based on the analysis of the binding energy position, the Cr element at the main peak of 574.60 eV mainly exists in the Cr–N chemically bound state, indicating that Cr participates in the formation of the stable nitride phase. Meanwhile, a slight tailing phenomenon can be observed on the high binding energy side, indicating the presence of a small amount of Cr–O bonds on the coating surface. This is mainly due to the slight oxidation of the surface layer of the sample after exposure to air, rather than the formation of a large amount of oxide inside the coating.

Figure 5d shows the high-resolution spectrum of W 4*f*, which also exhibits a distinct bimodal structure. The main peak of W 4*f*_7/2_ is located at 29.07 eV, corresponding to the characteristic binding energy of W^4+^. The main peak of W 4*f*_5/2_ is approximately 32 eV, and the spin splitting energy is about 3 eV, indicating that the W element mainly exists in the low-valent W combination state and forms W–N bonds with N. W^4+^ has excellent electronic conductivity. When combined with Cr^3+^, it can form bimetallic active sites, which is conducive to enhancing the comprehensive performance of the coating. Compared with pure metal W, its binding energy shows a certain shift, which indicates that the electron cloud density around W is jointly affected by the high-entropy alloying effect and the nitriding reaction. In addition, a weak oxidation peak was detected in the high binding energy region, indicating that there are a small number of W–O bonds on the sample surface, but their strength is significantly lower than that of the main peak, suggesting that the main structure of the coating is still dominated by nitrides.

Figure 5e shows the high-resolution spectrum of Nb3*d*, which exhibits a distinct dual-peak structure of Nb3*d*_5/2_ and Nb3*d*_3/2_. Based on the fitting results in the figure, Nb3*d*_5/2_ is located at approximately 207.5 eV, and Nb3*d*_3/2_ is located at approximately 204.3 eV. The binding energy positions correspond respectively to the two oxides of niobium, Nb_2_O_5_ and NbO_2_, indicating that there is a certain degree of oxidation of Nb on the coating surface. It should be particularly noted that since Ar^+^ etching was not performed in this test, the oxidized components with higher binding energy in Nb3*d* cannot be simply equated with the formation of a large amount of Nb_2_O_5_ inside the coating, but are more likely to reflect the natural oxidation of the outermost layer of the coating.

Figure 5f shows the high-resolution Mo3*d* spectrum, where distinct Mo3*d*_5/2_ and Mo3*d*_3/2_ bipeaks can be observed. The main components in the approximately 227–229 eV region correspond to the Mo–N chemical environment. This result indicates that Mo can react with active nitrogen and form Mo–N bonds during reactive sputtering, suggesting that the Mo element mainly participates in the composition of the high-entropy phase in the form of metal nitrides.

Figure 5g shows the high-resolution spectrum of Ta 4*f*,, featuring paired Ta 4*f*_5/2_ and Ta 4*f*_7/2_ peaks, indicating that Ta has typical 4*f* spin–orbit splitting characteristics, both corresponding to the Ta–N chemical binding state, suggesting that Ta is also involved in the nitriding reaction of the coating. Ta is a typical strong nitride-forming element. The Ta–N bond it forms with N has high bond energy and a stable structure, which can effectively enhance the bonding strength and thermal stability of the nitride phase. It is an important structural basis for coatings to have excellent mechanical properties and corrosion resistance.

Figure 5h shows the high-resolution spectrum of V2*p*. Two main peaks, V2*p*_3/2_ and V2*p*_1/2_, can be observed, located near approximately 513 eV and 520 eV, respectively, corresponding to the V–N bonding form. This indicates that the V element is also dissolved in the high-entropy nitride lattice in the form of vanadium nitride, further enriching the composition of the multi-component solid solution. Strengthen the structural stabilizing effect brought about by the high-entropy effect. The formation of V–N bonds can further alter the local electronic structure and lattice environment simultaneously, enabling the formation of complex multi-element coordination relationships among different transition metal elements. This is consistent with the lattice distortion effect caused by the coexistence of multiple elements in high-entropy nitrides.

Figure 5i shows the high-resolution spectrum of Si 2*p*, with its main peak located around 101.9 eV, corresponding to the Si–N bond. The low binding energy components at approximately 99.3 eV mainly correspond to the Me–Si bonding environment formed by Si and metal elements and may also contain a small amount of Si–Si-like chemical environments. The presence of the Si element has a significant regulatory effect on the microstructure of the coating. On the one hand, the existence of Si–N bonds further proves that Si can have a strong chemical combination with N during reactive sputtering. This may be conducive to the formation of stable amorphous/nanoscale Si–N enrichment regions and has a certain inhibitory effect on grain growth, refines the coating structure, and enhances the hardness and corrosion resistance of the coating. On the other hand, a small amount of the Me–Si phase is enriched at the grain boundaries, which can prevent dislocation slip and grain boundary migration, further enhancing the anti-plastic deformation ability of the coating, which is consistent with the mechanism of the Si element regulating the nanocrystalline composite structure.

Figure 5j shows the high-resolution spectrum of N1*s*. The spectrum mainly features a strong peak near approximately 397 eV, corresponding to the N–Me chemical environment. Since Cr, W, Nb, Mo, Ta and V all have certain nitride formation capabilities, this N1s peak actually reflects the superposition of multiple local metal–N coordination environments and should not be simply attributed to nitrides formed by a single element. Combined with the results of Cr2*p*, W4*f*, Mo3*d*, Ta4*f*, V2*p* and Si2*p*. It can be determined that the N element has formed stable chemical bonds with various metal elements, constituting multiple local coordination structures such as Cr–N, W–N, Nb–N, Mo–N, Ta–N, V–N and Si–N, and forming a stable high-entropy nitride skeleton.

In summary, the surface of the CrWNbTaMoVSiN high-entropy nanocomposite coatings prepared under −80V bias voltage is mainly composed of metal nitrides. Meanwhile, there are small amounts of oxides of elements such as Cr, W, and Nb on the surface, which are natural passivation layers produced by air exposure. Si simultaneously exhibits both Me–Si and Si–N chemical environments. This indicates that a complex chemical bonding network is formed during the reactive magnetron sputtering process, with multi-metal nitrides as the main body and Si–N/Me–Si local structures participating together. XPS analysis fully revealed the composite nanostructure characteristics of the coating, providing a solid material structure basis for the coating to have high hardness, good wear resistance and chemical stability.

### 3.4. The Influence of Substrate Bias Voltage on Coating Hardness

**Figure 6:**
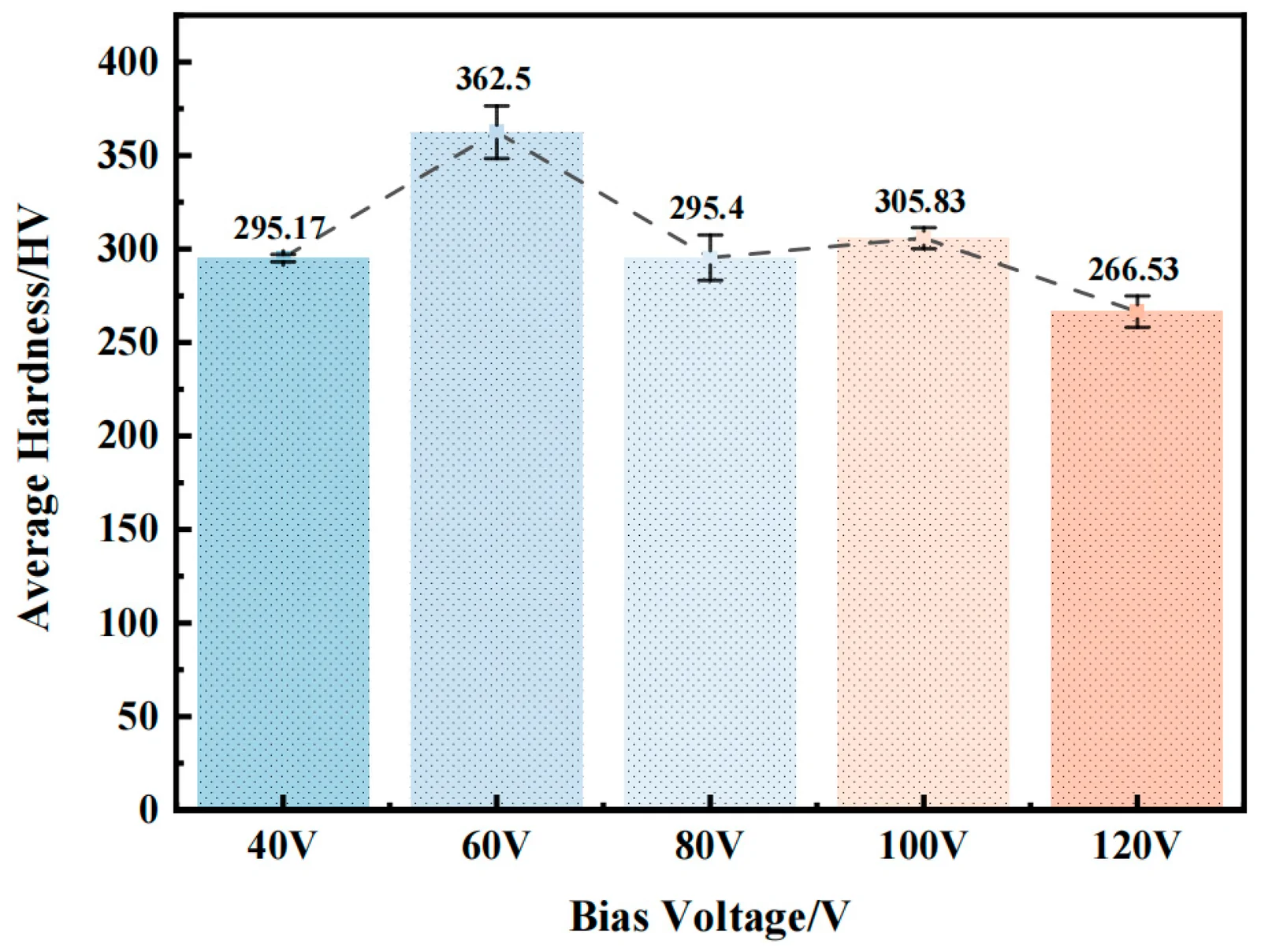
Bar chart of hardness of CrWTaMoVSiN high-entropy nano-multilayer coating under different bias voltage.

As shown in Figure 6, the hardness of the CrWNbTaMoVSiN high-entropy nitride coating shows a pattern of first increasing and then decreasing with the variation in bias voltage: when the substrate bias voltage increases from −40 V to −60 V, the average hardness of the coating significantly increases from 295.2 HV to 362.5 HV, reaching the peak hardness among all samples. When the bias voltage continues to increase from −60 V to −120 V, the hardness of the coating shows a continuous downward trend. At −120 V, the average hardness drops to 266.5 HV. The hardness of the coating is optimal under a bias voltage of −60 V. Possible reasons include effects of fine grain strengthening, solid solution strengthening, interface strengthening and structural densification. The moderately increased ion bombardment energy promotes the lateral migration of deposited atoms, effectively inhibits the growth of columnar crystals under low bias voltage, achieves grain refinement and increases the number of grain boundaries. Based on the Hall-Petch relationship, the hindrance effect of grain boundaries on dislocation slip and proliferation is significantly enhanced, bringing about a fine-grain strengthening effect in the core. Meanwhile, strong nitride-forming elements such as Cr, W, Nb, and Ta fully combine with N atoms under the action of ion bombardment, forming a large number of strongly bonded Me–N covalent bonds, intensifying the high-entropy lattice distortion, and generating a significant solid solution strengthening effect. The nanomultilayer structure with alternating deposition of metal layers and nitride layers forms a clear interlayer interface under this bias voltage, which can effectively block the cross-layer slip of dislocations. The interface strengthening effect further enhances the coating’s resistance to plastic deformation. In addition, moderate ion bombardment eliminates microscopic defects such as internal pores and pinholes in the coating, enhances the density of the coating, reduces stress concentration and premature plastic deformation during hardness testing, and ultimately achieves a significant increase in macroscopic hardness. When the substrate bias voltage exceeds −60 V, high-energy ion bombardment will cause significant damage to the microstructure of the coating. This not only greatly weakens the various strengthening effects of the coating but also introduces a large number of harmful microscopic defects. This is the core cause that leads to the continuous decline of coating hardness as the bias voltage increases. Intense ion bombardment can trigger a strong anti-sputtering effect, which not only reduces the coating thickness from 455 nm at −60 V to 353 nm at −120 V but also disrupts the clear interlayer interfaces of the nano-multilayer structure, causing mutual diffusion of interlayer elements and ultimately forming a blended interface structure. This greatly weakens the pinning and hindrance effects of the interface on dislocation slip. This rule is consistent with the research conclusion that interface blending causes mechanical property degradation in existing nano-multilayer coating systems. Meanwhile, excessively high ion bombardment energy will significantly enhance the migration ability of atoms on the substrate surface, causing the coating grains to abnormally coarken from 10~15 nm at −60 V to 20~30 nm under −100 to −120V conditions. According to the Hall-Petch grain boundary strengthening theory, grain coarsening will directly weaken the grain boundary strengthening effect and significantly reduce the coating’s resistance to plastic deformation. In addition, strong ion bombardment can introduce excessive residual internal stress within the coating and simultaneously induce microscopic defects such as micro-pores and micro-cracks. Such defects can become stress concentration sites, and under the action of external loads, they can easily induce premature plastic deformation or even cracking of the coating, directly reducing the effective load-bearing capacity of the coating. The desorption of N atoms caused by intense ion bombardment will lead to a reduction in the number of strong covalent Me–N bonds within the coating, further weakening the solid solution strengthening effect and ultimately intensifying the continuous decline in coating hardness.

### 3.5. The Influence of Substrate Bias Voltage on the Friction and Wear Performance of Coatings

**Figure 7:**
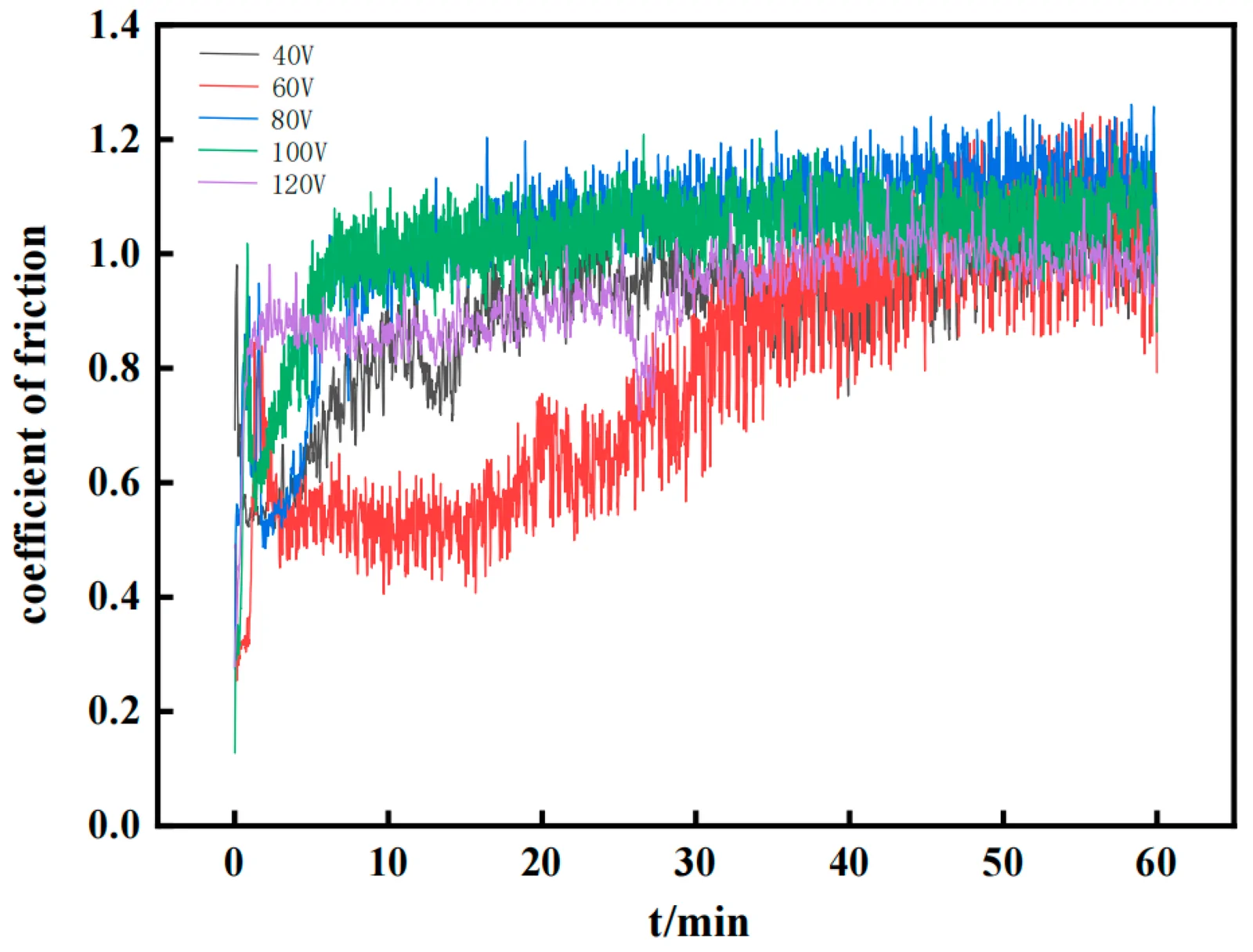
The coefficient of friction curves of CrWTaMoVSiN high-entropy nano-multilayer coating under different substrate bias voltage.

Figure 7 shows the coefficient of friction curves of CrWNbTaMoVSiN under different bias voltages. When the bias voltage is −40 V, the coating shows a relatively short running-in period. The coefficient of friction rises rapidly in the initial stage and eventually stabilizes around the average coefficient of friction of 0.895. Under the bias voltage of −60 V, the coating has a distinct running-in period. The coefficient of friction gradually increases over time and tends to stabilize after about 30 min. The average coefficient of friction is approximately 0.778, which is the lowest value under all bias voltage conditions. Under the bias voltages of −80 V and −100 V, the running-in period of the coating was not obvious. The coefficient of friction rose rapidly and remained at a relatively high level overall, with the average coefficients of friction being 1.038 and 1.011, respectively. The running-in period of the −120 V bias coating is relatively short, and the coefficient of friction stabilizes at an average coefficient of friction of 0.931 after increasing over time. It is worth noting that under high bias conditions, the coefficient of friction is significantly higher, and the stable value is larger. It is speculated that the mechanism is as follows: at the initial stage of friction, there may be a thin oxide film on the surface of the coating. After this oxide film is rapidly worn off, the high-entropy coating is directly exposed. Subsequently, under continuous reciprocating friction, the coating is gradually worn through locally, forming deep and continuous wear grooves. When the ceramic ball passes through this groove, the edges on both sides of the groove will exert additional plowing resistance and extrusion deformation resistance on it, resulting in a significant increase in frictional resistance, which is manifested as the coefficient of friction remaining at a high level continuously. Bias voltage has a significant impact on the friction stability, running-in behavior and failure mechanism of CrWNbTaMoVSiN coatings. Moderate bias voltage helps to reduce the steady-state coefficient of friction and prolong the running-in process, while too low or too high bias voltage is not conducive to the formation of a stable low-friction interface.

**Figure 8:**
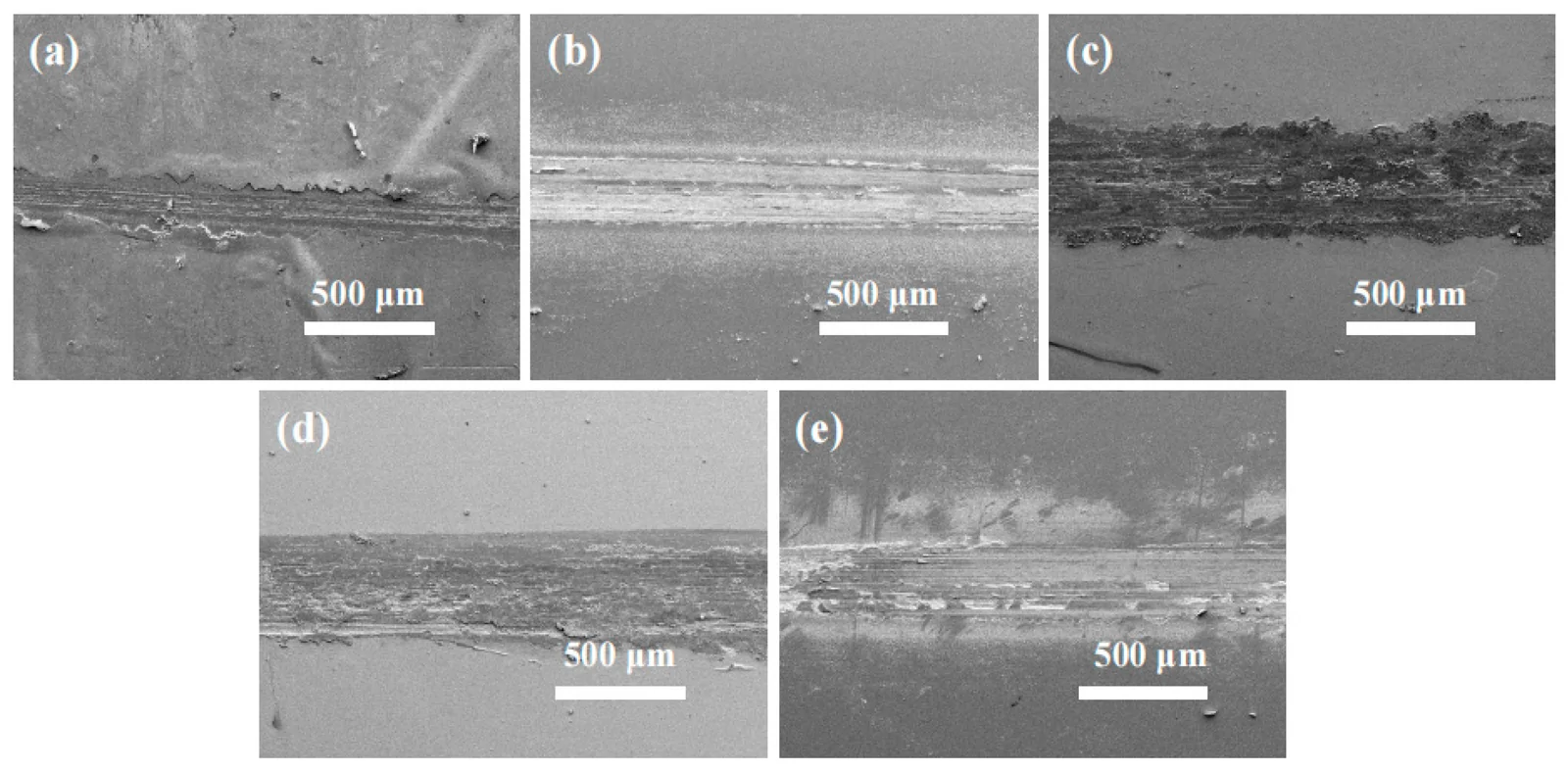
SEM morphology of coating wear marks under different bias voltage conditions. (**a**) −40 V. (**b**) −60 V. (**c**) −80 V. (**d**) −100 V. (**e**) −120 V.

Figure 8 shows the SEM morphology of the coating wear marks under different bias voltage conditions. Figure 8a–e represent the coatings at −40 V, −60 V, −80 V, −100 V, and −120 V, respectively. It can be observed from Figure 8a that at a bias voltage of −40 V, local interlayer peeling occurs near the wear trajectory, but no large-scale failure takes place. The wear mark width is narrow, and the damage is relatively light. At a bias voltage of −60 V, it can be observed from Figure 8b that during the friction process, abrasive wear and plowing wear are the main types. There are slight plowing grooves near the wear trajectory without obvious accumulation of grinding swarf. The coating was not damaged, and the grinding marks were uniform without large-scale failure. As the bias voltage increases from −80 V to −120 V, it can be observed through Figure 8c–e that the coating undergoes large-scale layered peeling and adhesive wear, with deep and dense furrows piled up near the wear trajectory, indicating severe failure of the coating. Low bias voltage will result in insufficient adhesion of the coating, which can only resist mild wear and cause local peeling. In contrast, high bias voltage has excessive energy, leading to excessive internal stress, increased brittleness of the coating, and large-area layered peeling, thus causing failure. The anode layer ion source can provide additional controllable ion flux and ion energy, promoting the migration of deposited particles and coating densification, thereby enhancing the regulatory effect of bias voltage on the microstructure and properties of the coating [35]. In this study, the hardness showed a trend of first increasing and then decreasing with the increase in bias voltage, reaching 362.5 HV at −60 V. This indicates that moderate ion bombardment is conducive to fine crystallization, structural densification and interface strengthening, while excessively high bias voltage leads to a decrease in hardness due to heavy sputtering, grain coarsening and defect accumulation. This variation pattern is consistent with the research results that in existing HEAN coatings, an increase in bias voltage promotes structural densification and hardness improvement, while excessively high bias voltage leads to performance degradation [36,37].

### 3.6. The Influence of Substrate Bias Voltage on the Corrosion Resistance of Coatings

To evaluate the corrosion resistance of CrWNbTaMoVSiN high-entropy nanocomposite coatings in artificial seawater under different substrate bias conditions, a 60 min open-circuit potential test was first conducted. After the system potential stabilized, the final open-circuit potential value was recorded, and then this stable open-circuit potential was used as the initial potential. The samples prepared at bias voltages of −40 V, −60 V, −80 V, −100 V and −120 V were systematically characterized by electrochemical impedance spectroscopy (EIS) and potentiodynamic polarization (Tafel) tests.

#### 3.6.1. Electrochemical Impedance Spectroscopy Analysis

**Figure 9:**
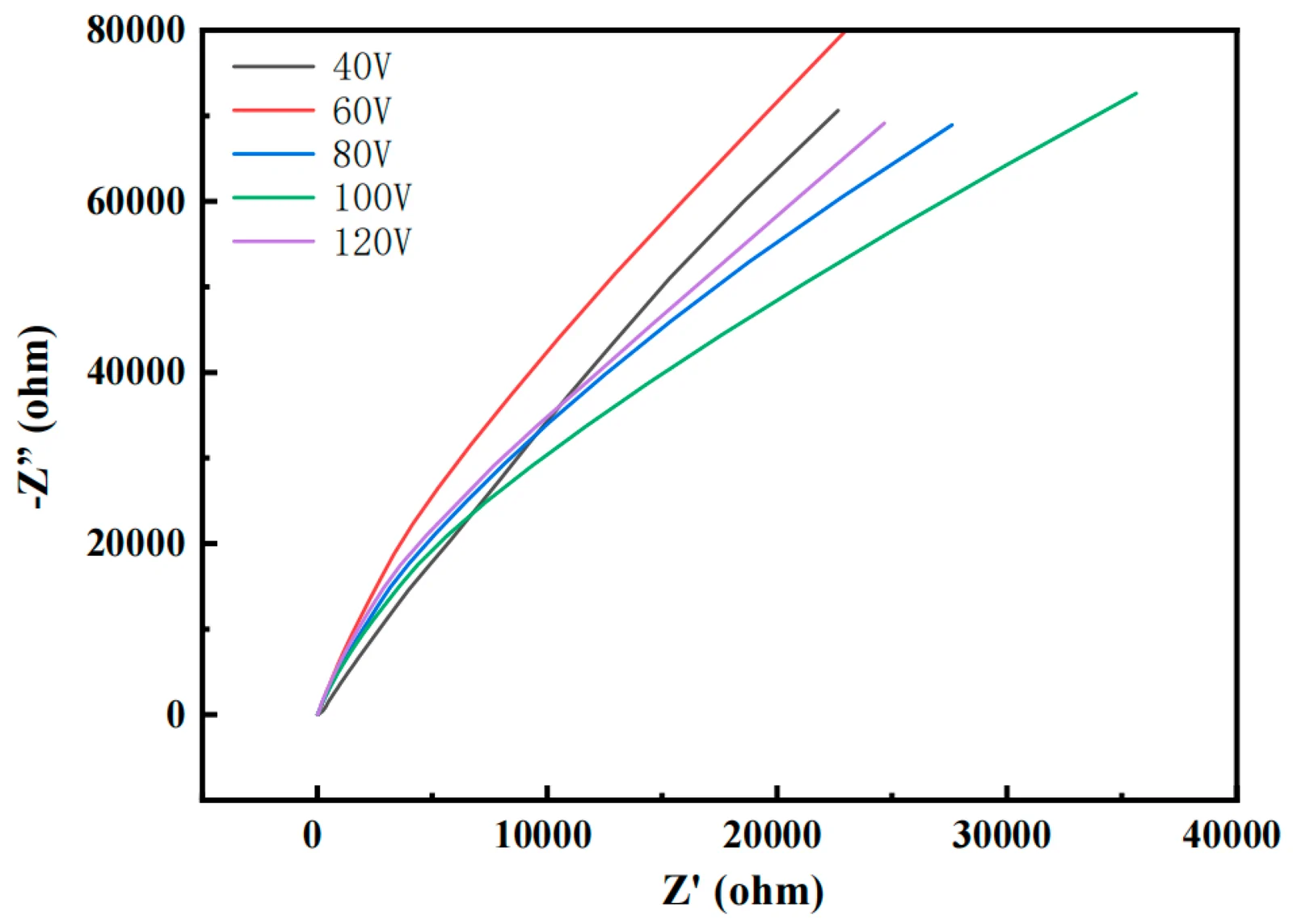
The complex impedance graph of the coating in artificial seawater under different substrate bias voltages.

Figure 9 shows the complex impedance graph of the CrWNbTaMoVSiN high-entropy composite nano-coating in artificial seawater under different substrate bias voltages. All samples exhibit impedance characteristics dominated by capacitive reactance arcs, indicating that the corrosion process is mainly controlled by interface charge transfer and coating barrier behavior. There are significant differences in the impedance arc radius under different bias voltages. Among them, the capacitive reactance arc radius of the −60 V sample is the largest, indicating that its interface charge transfer resistance is the highest, the corrosion reaction kinetics are the slowest, and the corrosion resistance is the best. The arc radius of the capacitive reactance of the −40 V sample is relatively small, indicating that the corrosive medium can more easily pass through the film layer defects and enter the interface area. When the bias voltage rises to −80 V, −100 V and −120 V, the impedance arc radius is reduced compared with −60 V, indicating that excessively high bias voltage does not further enhance corrosion resistance; instead, it may reduce the protective performance due to structural damage or residual stress effects.

#### 3.6.2. Kinetic Potential Polarization Analysis

**Figure 10:**
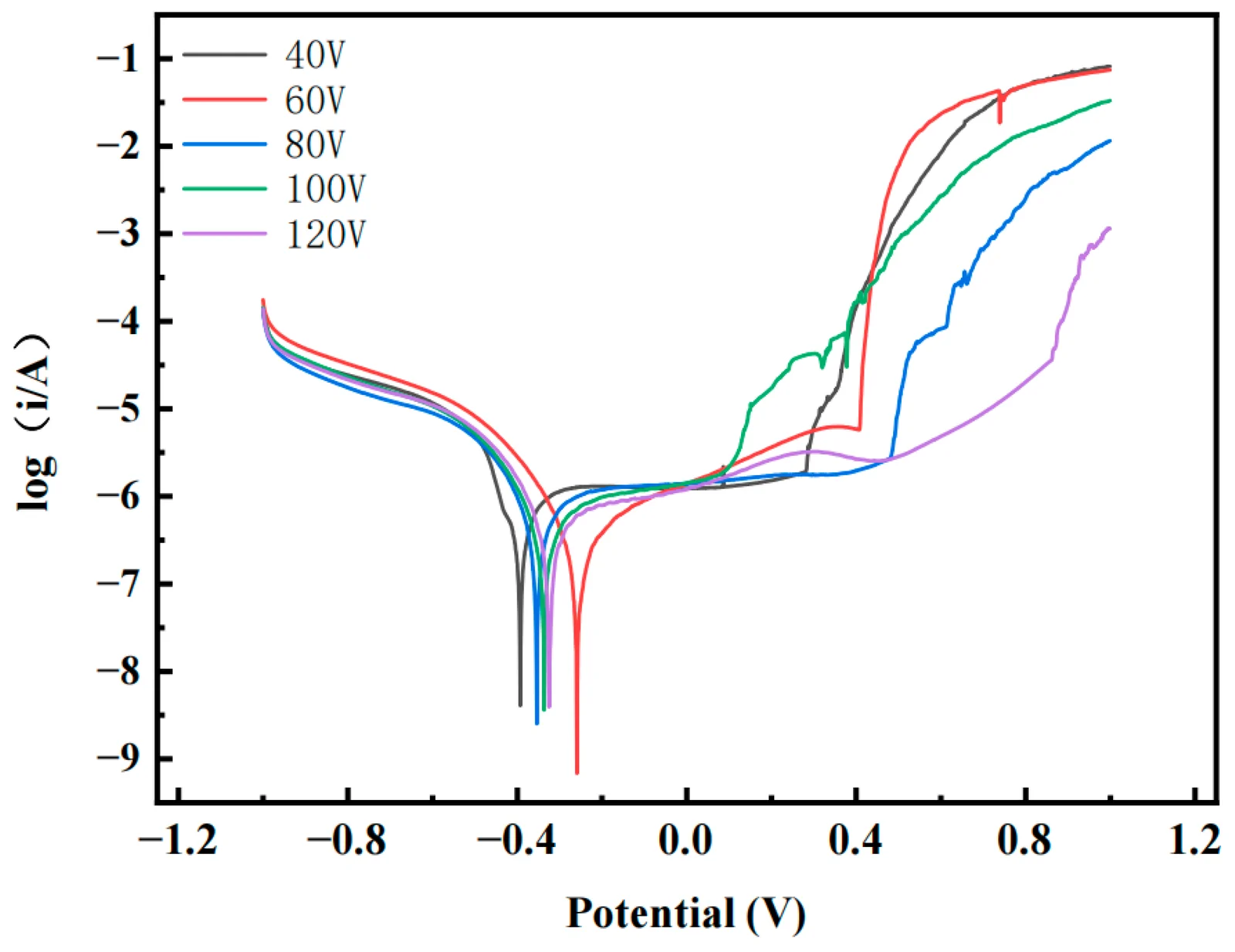
The kinetic potential polarization curves of samples with different substrate bias voltages in artificial seawater.

Figure 10 shows the potentiodynamic polarization curves of samples with different substrate bias voltages in artificial seawater. The cathode and anode branches of each sample all show significant differences, indicating that the substrate bias voltage has a significant regulatory effect on the corrosion reaction kinetics behavior of the coating. The more positive the self-corrosion potential Ecorr is, the lower the thermodynamic tendency of the material to corrode. The lower the self-corrosion current density (Icorr), the slower the corrosion kinetics rate and the better the corrosion resistance. Combined with the Tafel curve, it can be seen that the polarization curve of the −60 V sample as a whole shifts towards a lower current density, with the self-corrosion potential moving relatively positively and the self-corrosion current density being the smallest, indicating that its corrosion rate is the lowest and its corrosion resistance performance is the best.

The −40 V sample exhibits a higher anodic dissolution tendency and a greater corrosion current density, indicating that the coating prepared at a low bias voltage is difficult to form a sufficiently dense and continuous protective layer and is more prone to local activation dissolution in the Cl^−^ medium. As the bias voltage increases to −60 V, moderate ion bombardment enhances the surface migration ability of the deposited particles, and promotes the densification of the film layer and the strengthening of interface bonding, making it difficult for corrosive media to quickly reach the substrate surface through pores, grain boundaries and interlayer defects. Therefore, Icorr is significantly reduced. After further increasing the bias voltage to −80 V, −100 V and −120 V, the polarization current showed an upward trend again, indicating that an excessively high bias voltage is not conducive to maintaining the optimal corrosion resistance state. This is mainly attributed to the heavy sputtering effect caused by the continuous bombardment of high-energy particles, the regeneration of local defects, and the excessive accumulation of residual compressive stress, which leads to the formation of more complex corrosion channels in the film layer and weakens its electrochemical protective effect.

## 4. Conclusions

In summary, we have fabricated a high-entropy nanocomposite coating of CrWNbTaMoVSiN with alternating modulation of metals and nitrides by using an anode layer ion source-assisted magnetron sputtering. With the increase in bias voltage, the enhanced anti-sputtering effect of high-energy ion bombardment causes the coating deposition rate to continuously decline, and the thickness linearly decreases from 538 nm to 353 nm. The microhardness of the coating shows a single-peak variation characteristic of first increasing and then decreasing with the substrate bias voltage. The significant increase in hardness under moderate bias voltage is attributed to the synergistic effect of fine-grained strengthening, solid solution strengthening, and nano-multilayer interface strengthening. The electrochemical test results further indicate that the substrate bias voltage has a significant regulatory effect on the corrosion resistance behavior of the coating in artificial seawater. The −60 V sample exhibits the largest capacitive reactance arc radius, a higher low-frequency impedance value, and a lower corrosion current density, suggesting that it has the optimal interface charge transfer impedance and dielectric shielding capability. This paper systematically studies the influence of bias voltage on the structure and performance of seven-element high-entropy nano-multilayer coatings containing Si, providing an experimental basis for the application of high-entropy composite nano-coatings in marine environments.

## Data Availability

All data generated or analyzed during this study are fully included in this published article.
